# Smoking-Dependent Association of Serum Brain-Derived Neurotrophic Factor with Pulmonary Function Parameters in Chronic Obstructive Pulmonary Disease

**DOI:** 10.3390/medicina60071111

**Published:** 2024-07-09

**Authors:** Elina Aleksandrova, Dimo Dimov, Tanya Tacheva, Hristina Petrova, Kahan Celik, Tatyana Vlaykova

**Affiliations:** 1 Department of Medical Chemistry and Biochemistry, Medical Faculty, Trakia University, 6000 Stara Zagora, Bulgaria; dimo.dimov@trakia-uni.bg (D.D.); tanya.tacheva@trakia-uni.bg (T.T.); hristina.petrova@trakia-uni.bg (H.P.); kahan.celik.20@trakia-uni.bg (K.C.); tatyana.vlaykova@trakia-uni.bg (T.V.); 2 Department of Medical Biochemistry, Faculty of Pharmacy, Medical University of Plovdiv, 4000 Plovdiv, Bulgaria

**Keywords:** BDNF, COPD, serum, ELISA, spirometry

## Abstract

*Background and Objectives*: One of the members of the neurotrophin (NT) family is the brain-derived neurotrophic factor (BDNF). In addition to its role in the nerve system, it has been found to play a role in lung health and diseases. *Materials and Methods*: The serum concentrations of BDNF were assessed in 57 patients with COPD and in 19 control individuals and the possible associations of BDNF with the spirometric indexes and disease stages were explored. *Results*: We did not find a significant difference between the serum concentrations of BDNF of patients and controls (*p* = 0.521). A significant negative correlation of the serum BDNF levels with the age of the patients (Rho = −0.279, *p* = 0.036) was observed. In addition, a borderline negative correlation with the age of disease onset (Rho= −0.244, *p* = 0.063) was also found. When analyzing these correlations in different genders, we found stronger statistical significance in male patients (Rho = −0.398, *p* = 0.009; and Rho = −0.419, *p* = 0.006), while no such significance was found in females (*p* = 0.574 and *p* = 0.342). The analyses of the possible relations of serum BDNF concentration with the spirometric parameters in the whole group of patients did not reveal any significance (*p* = 0.231 for FEV1%pr. and *p* = 0.271 for FEV1/FVC%). However, when the patients were dichotomized on the basis of smoking habits, we obtained a strong positive correlation between BDNF and FEV1%pr. (Rho = 0.501, *p* = 0.048) in non-smokers, but strong negative correlations with FEV1%pr. (Rho = −0.468, *p* = 0.003) and with FEV1/FVC% (Rho = −0.331, *p* = 0.040) in ex/current smokers. Non-smokers with moderate disease (GOLD II) had higher BDNF serum concentrations than patients with GOLD stage III/IV (*p* = 0.031). In ex/current smokers, there was an opposite association (*p* = 0.045). *Conclusions*: The results of our study suggest that the expression and secretion of BDNF are changed in COPD, but its effects and functions may differ according to the smoking history of the patients.

## 1. Introduction

The brain-derived neurotrophic factor (BDNF) is a member of the neurotrophins (NTs), a family of four polypeptides similar in structure. BDNF was first identified as a soluble factor maintaining the survival of sensory neurons [1]. It has also been involved in the differentiation and growth of neurons, protecting them from apoptosis and stimulating their regeneration [2]. In the nervous system, BDNF is considered to control its own expression and activity in an autocrine manner, activating a variety of downstream signaling cascades, including phospholipase C (PLC), phosphatidylinositol 3 kinase (PI3K), mitogen-activated protein kinases (MAPKs), and nuclear factor kappa-light-chain-enhancer of activated B cells (NFκB) [3].

The BDNF gene has been mapped on chromosome 11p13-14 of the human genome. Its expression is initiated by several promoters producing different transcripts, some of which are brain-specific [4]. The secretion of the BDNF starts in the endoplasmic reticulum of the source cells as a precursor molecule (pre-pro BDNF), which is further processed in the Golgi apparatus to proBDNF. The mature BDNF (mBDNF) is formed either intracellularly or extracellularly [5,6]. The proBDNF peptide and its mature form are neuroactive but exert opposite functions. While ProBDNF is found to stimulate apoptosis through the common neurotrophin receptor p75NTR, a member of the tumor necrosis factor receptor family (TNFR), mBDNF binds with high affinity to the Tropomycin receptor kinase B (TrkB), supporting cell survival [7]. Thus, the proBDNF/mBDNF ratio plays a significant role in neuronal cells’ stability [8].

Although generally considered as a neuroprotector, BDNF has also been involved in the cellular energy homeostasis and in the inflammation processes. Besides being in the nervous system, it has been detected in a large variety of non-neuronal tissues and cells. The sources of peripheral BDNF are several tissues including the liver, lung, muscles, spleen, and vascular smooth muscles [9]. Previous studies have reported that circulating BDNF could also be produced from lymphocytes, mast cells, macrophages, and other immune cells [10,11], which confirms its possible role as a modulator linking the neuronal tissue with the immune system. The produced BDNF remains stored in blood platelets [12] and is selectively released during platelet activation [13] as the pro- or the mature form.

Recently, the implication of BDNF in human lung disease has been discussed. Evidence from murine studies suggests that the bronchial epithelium secretes BDNF in the lungs, especially after allergen exposure [14], revealing its role in airway remodeling and function [15]. In human lungs, increased BDNF levels have been observed in sputum and bronchoalveolar lavage (BAL) fluid from patients with asthma [15]. Furthermore, elevated serum BDNF concentrations have been reported to correlate with disease severity [16]. The effects of BDNF gene polymorphisms on asthma susceptibility and severity have also been addressed and a possible association of BDNF gene variants with asthma severity has been reported [17,18]. 

A few studies have focused on the role of BDNF in chronic obstructive pulmonary disease (COPD)-related airway inflammation. The pathogenesis of COPD includes persistent inflammation and subsequent remodeling of the lower airways and lung parenchyma, causing impairment of the lung function. COPD prevalence is increasing globally and the habit of cigarette smoking is a first-line predisposing factor. Recent studies on a rat model of COPD suggest that cigarette smoking stimulates BDNF expression in the airway and anti-BDNF treatment may affect airway inflammation and lung function [19]. Also, evidence indicating that BDNF is an important biomarker associated with parameters of COPD severity has been reported [20].

Currently, no therapeutic approaches have been found to be sufficiently effective in preventing the progression of COPD, probably due to the heterogeneous clinical and molecular nature of the disease and the lack of reliable biomarkers targeting many of its characteristics. Thus, we aimed to evaluate the serum BDNF levels in a group of patients with COPD from the Bulgarian population and to investigate the association of serum BDNF with pulmonary function parameters in the studied group. 

## 2. Materials and Methods

### 2.1. Study Subjects

A group of 57 patients diagnosed with COPD and 19 control individuals were included in the present study. The patients’ group consisted of 42 male and 15 female individuals with a mean age of 66.76 (±1.43, SEM) and 68.20 (±3.00, SEM), respectively. The patients were recruited by specialists–pulmonologists from the Clinic of Internal Medicine, University Hospital, Trakia University, Stara Zagora, Bulgaria. The inclusion criteria for enrolling the patients in this study were those as described by Tacheva et al., 2021 [21]: age higher than 40 years; forced expiratory volume in 1 s (FEV1) of <80%; forced expiratory volume in 1 s (FEV1)/forced vital capacity (FVC) ratio of <70%; and FEV1 reversibility after the inhalation of Salbutamol at 400 mg of <12%. 

The control group included 8 male and 11 female subjects, non-affected by inflammatory lung diseases or any other chronic inflammatory or cancer diseases. Written informed consent was obtained from each individual prior to this study. This work was approved by the Ethics Committee of Medical Faculty, Trakia University, Stara Zagora, Bulgaria, protocol number 16/19, March 2021.

### 2.2. Clinical Data Collection

Demographic and clinical data from all subjects were collected, including age, sex, course of disease, smoking status, and smoking habits (number of cigarette packs/year). Demographic and clinical parameters are summarized in Table 1. 

### 2.3. Spirometric Analysis

The lung function of the patients was assessed by applying the fast spirometry according to the method reported by Quinter using a spirometer Pony FX(Cosmed, Rome, Italy) as described earlier [22]. The main analyzed spirometric indexes, FEV1 (forced expiratory volume in one second) as a percentage of the predicted value and the ratio of FEV1 to the forced vital capacity (FVC) (FEV1/FVC%), were compared to the normal values: between 80 and 120% for FEV1% predicted and more than 0.70 for FEV1/FVC. 

In our study, we applied the COPD staging according to GOLD (Global strategy for the diagnosis, management, and prevention of COPD) 2017, which is based on the airflow limitation in patients with FEV1/FVC < 0.70 (70%): GOLD I is mild—FEV1 ≥ 80% predicted; GOLD II is moderate—50% ≤ FEV1 < 80% predicted; GOLD III is severe—30% ≤ FEV1 < 50% predicted; and GOLD IV is very severe—FEV1 < 30% predicted [23].

### 2.4. Quantification of Serum BDNF Concentration

Venous blood (2 mL) was collected from patients diagnosed with COPD and healthy individuals. The serum was extracted after removing the blood cloth by centrifuging the samples at 3500 rpm for 5 min and was further stored in aliquots at −20 °C until the assay was conducted. The enzyme-linked immunosorbent assay (ELISA) was used for BDNF concentration measurement with a commercially available kit (SunRed, Biotechnology Company, Shanghai, China). The optical density (OD) at 450 nm was used for assessing the results. A standard curve constructed with the kit’s standards was used to determine the cytokine concentration, expressed in picograms per mL (pg/mL). Serum samples of patients and controls were analyzed together in the same analytic batch. The detection range of the BDNF ELISA kit was 100–18,000 pg/mL and sensitivity was 75.85 pg/mL.

### 2.5. Statistical Methods

SPSS, 16.0 for Windows (SPSS Inc., Chicago, IL, USA) was used for performing the statistical analysis. Two tests, Kolmogorov–Smirnov and Shapiro–Wilk tests, were applied for assessing the distribution of the continuous variables. If the continuous variables were with normal distribution, the Student’s *t* test and analysis of variance (ANOVA) test LSD post hoc were applied for comparing the data between independent groups, while when the variables were with non-normal distribution, the Mann–Whitney U test and Kruskal–Wallis test were applied. Correlations were assessed by Pearson or Spearman’s test depending on the normality of the continuous variables. Factors with *p* < 0.05 were considered statistically significant. 

## 3. Results

**Figure 1:**
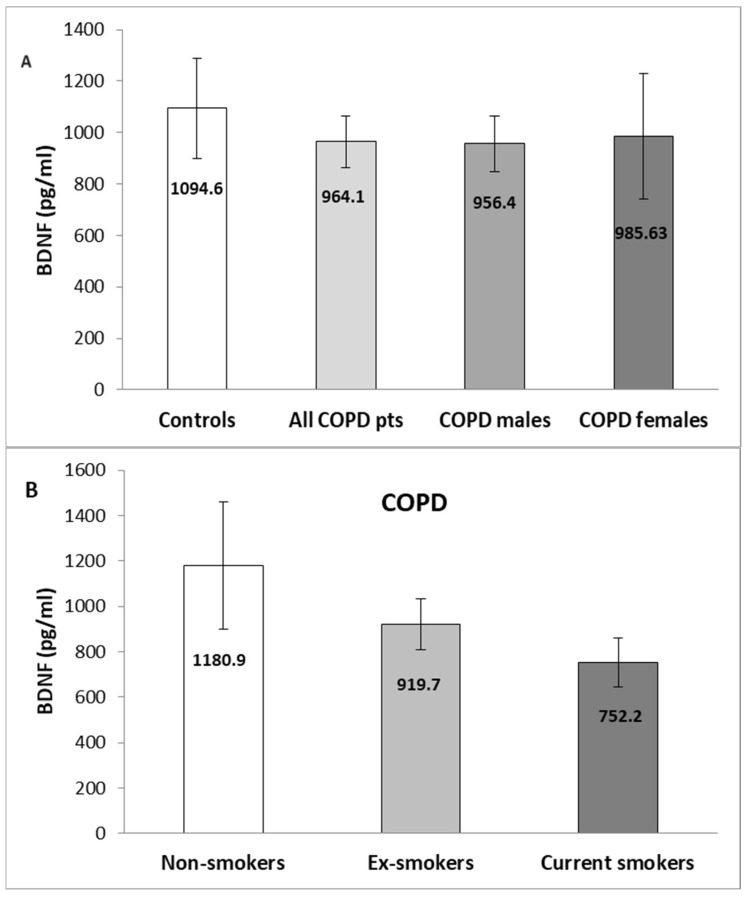
(**A**) Serum levels of BDNF in controls vs. patients with COPD and genders and (**B**) in COPD cases stratified by smoking status. Values are expressed as mean (±SEM) in pg/mL.

Although we did not find a significant difference in BDNF serum levels in patients with COPD compared to controls, we observed lower concentrations in the cases with a mean value of 964.1 ± 101.0 (SEM) pg/mL (median of 832.5 pg/mL, range of 190.7–4114.1 pg/mL) and 1094.6 pg/mL ± 194.5 (SEM) pg/mL (median of 845.7 pg/mL, range of 395.2–3637.0 pg/mL) of the control group (*p* = 0.521, Mann–Whitney U test) (Figure 1A). When we analyzed the patients’ group, we did not observe differences in serum BDNF between the genders (*p* = 0.697, Mann–Whitney U test) (Figure 1A) and smoking habits (*p* = 0.846, Kruskal–Wallis test) (Figure 1B). 

**Figure 2:**
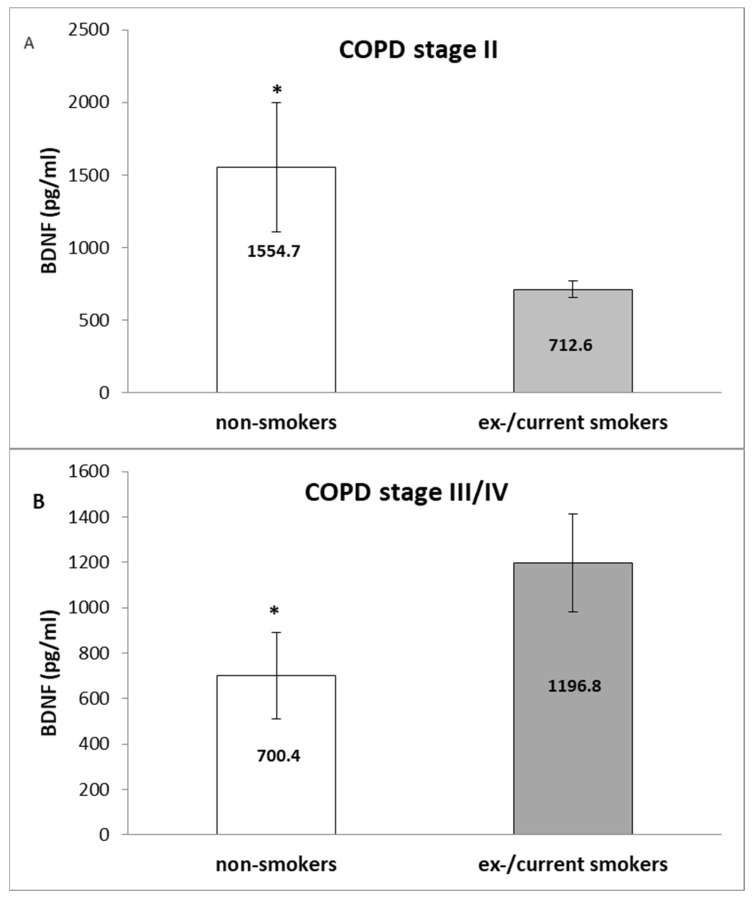
Association of BDNF serum levels with smoking habits in patients with moderate COPD (GOLD stage II) (**A**) and in patients with severe/very severe disease (GOLD stage III/IV) (**B**). Values are expressed as mean (±SEM) in pg/mL. Statistically significant values are presented with *.

However, when the patients were stratified according to the severity of the disease to cases with moderate disease (stage II, *n* = 36) and with severe/very severe COPD (stage III/IV, *n* = 21), statistically significant results were obtained (Figure 2): in stage II, the non-smokers had significantly higher serum BDNF than the ex/current smokers (*p* = 0.026, Mann–Whitney U test) (Figure 2A). Opposite association was seen in patients with severe/very severe disease (stage III/IV) (*p* = 0.046, Mann–Whitney U test) (Figure 2B). 

However, no correlations were observed between the serum levels of BDNF and the intensity of smoking (packs/year) either in the whole group of smokers (ex- and current smokers all together, Rho = 0.054, *p* = 0.745) or in the separate groups of smokers: current (Rho = −0.472, *p* = 0.199) and ex-smokers (Rho = 0.217, *p* = 0.250). In addition, we did not find correlations between the levels of BDNF and intensity of smoking when patients were divided according to the gender (Rho = 0.053, *p* = 0.769 in males and Rho = −0.530, *p* = 0.280 for females) or GOLD stages (Rho = −0.089, *p* = 0.666 for stage II and Rho = −0.072, *p* = 0.816 for stages III/IV). 

**Figure 3:**
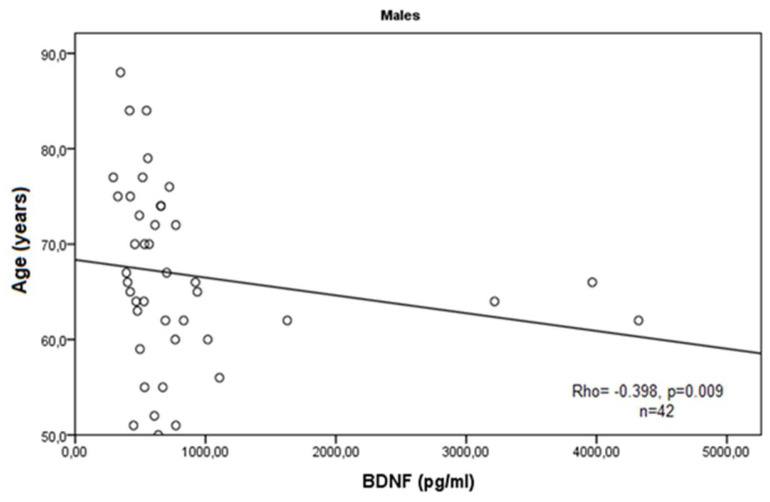
Correlation of BDNF serum levels expressed in pg/mL with age of male patients with COPD.

**Figure 4:**
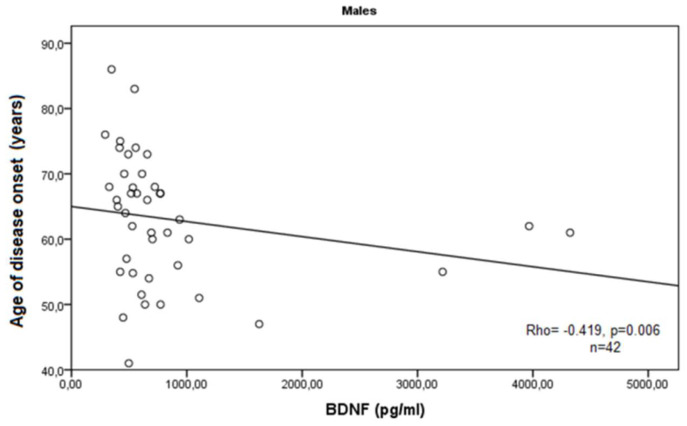
Correlation of BDNF serum levels expressed in pg/mL with age of disease onset in male patients with COPD.

According to the demographic and clinical data of the COPD cases, we observed a statistically significant negative correlation of BDNF serum levels with the age of enrollment of the patients in this study (Rho = −0.279, *p* = 0.036, Spearman correlation test), and a marginal negative correlation with the age of disease onset (Rho = −0.244, *p* = 0.063, Spearman correlation test). In male patients, these findings were more obvious (Rho = −0.398, *p* = 0.009; and Rho = −0.419, *p* = 0.006) (Figure 3 and Figure 4).

**Figure 5:**
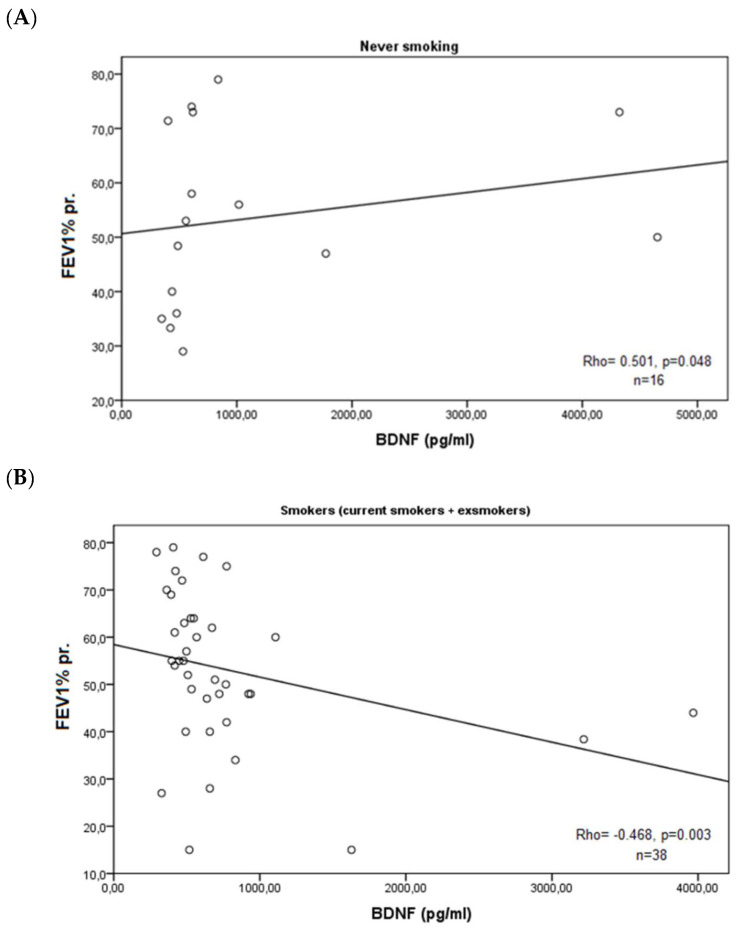
Correlations of BDNF serum levels and predicted forced expiratory volume (FEV1% pr.) in non-smoking patients with COPD (**A**) and in current/ex-smokers with COPD (**B**).

**Figure 6:**
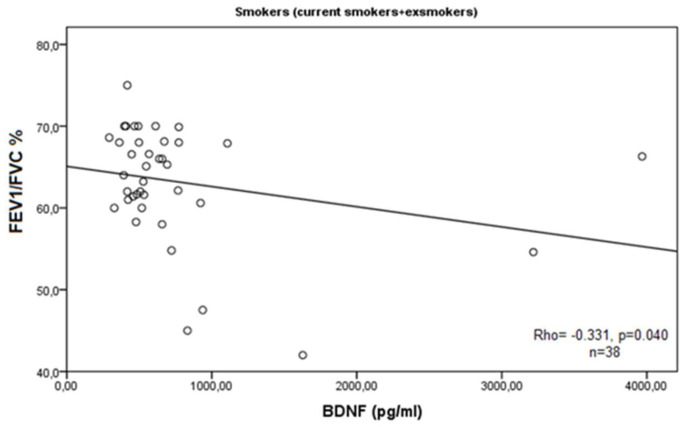
Correlation of BDNF serum levels and lung function parameter (FEV1/FVC%) in current/ex-smokers with COPD.

In the whole group of patients, there were no correlations between measured BDNF and the lung function indexes (*p* = 0.231 for FEV1%pr. and *p* = 0.271 for FEV1/FVC%). When we grouped the cases according to smoking habits (never smoking vs. ex/current smokers), we found that in non-smokers’ group (*n* = 16), there was a strong positive correlation between BDNF and FEV1%pr. (Rho = 0.501, *p* = 0.048) (Figure 5A), while in ex/current smokers (*n* = 38), there were statistically significant negative correlations with FEV1%pr. (Rho = −0.468, *p* = 0.003) (Figure 5B) and with FEV1/FVC% (Rho = −0.331, *p* = 0.040) (Figure 6).

**Figure 7:**
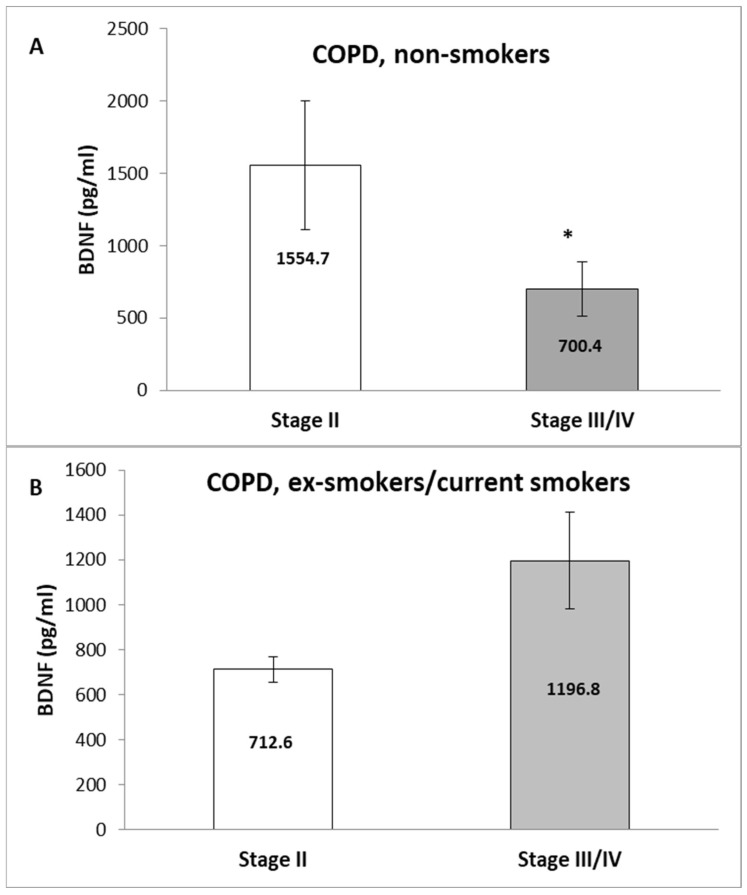
Association of BDNF serum levels and GOLD stage of patients with COPD. (**A**) Non-smokers and (**B**) ex/current smokers. Values are expressed as mean (±SEM) in pg/mL; statistical significance is marked with *.

Notably, the never-smoking patients with a moderate disease stage (GOLD stage II) had increased BDNF compared to patients with advanced disease (stage III/IV) (1513.6 ± 565.7 vs. 639.6 ± 190.0 pg/mL, *p* = 0.031, Mann–Whitney U test) (Figure 7A), while an opposite association was seen in the ex/current smoker group of patients (552.1 ± 36.8 vs. 1134.6 ± 292.3 pg/mL, *p* = 0.045, Mann–Whitney U test) (Figure 7B).

## 4. Discussion

COPD is an inflammatory lung disease with increasing prevalence worldwide [24]. A number of risk factors have been shown to contribute to the inflammatory lung condition of COPD including smoking and indoor and outdoor air pollution [25]. The systemic manifestations of COPD with the associated cardiovascular risk and respiratory failure have attracted scientific interest in identifying circulating biomarkers in these patients [26]. A role for BDNF in airway inflammation, remodeling, and hyperactivity has been suggested based on animal studies [27]. Airway and alveolar remodeling has not been efficiently achieved by current pharmacotherapy of COPD and the underlying mechanisms of this disease remain unclear [28]. 

As a neurotrophin closely related to tissue remodeling during chronic inflammation, BDNF is a promising target in COPD research. A pilot study measuring the serum levels of a large number of inflammation-related markers reported that BDNF is among the most elevated mediators in COPD [26]. Contrary to these findings, in our study, we did not find a significant difference in serum BDNF concentrations in a cohort of 57 patients with COPD compared to healthy individuals. In our patient group, we observed lower BDNF in sera, which can be explained with the interference of other immune factors on the production of neurotrophins. It has been well described that strong inflammatory conditions reduce BDNF expression due to the dysregulation of cytokine and other chemoattractants’ background [29]. Inflammation-related BDNF deficiency could underlie the impaired tissue remodeling in the airways combined with a variety of other factors such as environmental, demographic, or genetic factors [30]. One obvious demographic aspect in COPD pathogenesis is the older age of the patients, which is a predisposition for prolonged exposure to environmental detriments, stress, and age-related down-regulation of tissue repair and remodeling. Accordingly, we report a significant negative correlation of BDNF serum levels with the age of enrollment of the patients in this study (*p* = 0.036) and with the age of disease onset (*p* = 0.063), especially in male patients with COPD (*p* = 0.009 and *p* = 0.006, respectively). As a chronic disease, it is evident that advanced age contributes to COPD progression and our findings suggest that BDNF serum levels are decreasing in older patients with COPD. 

Taking into account that cigarette smoking is a major risk factor for COPD, we analyzed our group of patients in relation to lung function parameters and smoking habits. A variety of confounding factors are involved in the BDNF expression in smokers. One important note is that platelets, which are the repository of secreted BDNF, are highly influenced by the coagulation process in smokers [31]. Additionally, airway tissue itself could be a source of peripheral BDNF following cigarette smoking-related oxidative stress [32]. Also, serum BDNF has been reported to depend on the amount and duration of smoking, in smokers with anxiety and/or depression, but without any other reported diseases, it was found that a higher number of smoking years relates to higher serum BDNF [33]. In our group of patients with COPD, we did not find such correlation between the intensity of smoking (packs/year) and BDNF serum levels, which might be due to the presence of the chronic inflammation and the accompanying oxidative stress, which confound the secretion of this regulatory polypeptide. This notion can be supported by the observed differences in the levels of BDNF between patients with different smoking habits with mild COPD disease (GOLD II), where a lower level of oxidative stress is expected, and in opposition, in patients with severe/very severe COPD, where the oxidative stress is usually stronger [34].

Previously, data from a microarray analysis of a large number of serum markers indicated that BDNF is the strongest predictor of reduced forced expiratory volume in 1 s (FEV1) in COPD [16]. We observed significant positive correlation between the predicted forced expiratory volume (FEV1%) and serum BDNF levels in the non-smokers’ group and a strong negative correlation in the current/ex-smokers’ group. Our results support the notion that BDNF may modulate airway remodeling and epithelium-derived bronchodilator responses. As a neurotrophin growth factor, probably expressed from local tissues or nerves themselves, BDNF can promote the survival and development of sensory neurons [35], which innervate airway smooth muscles and the mucous cells. In the lungs, BDNF is also known to be additionally produced, especially after cigarette smoke exposure, by structural cells, as smooth muscle cells. The proposed hypothesis is that nicotine exposure stimulates the expression of both TrkB and p75NTR, and BDNF works via an autocrine mechanism to increase cell proliferation and airway contractility [27,36]. BDNF can also influence the expression levels of other factors, thus enhancing the bronchoconstriction [37]. It has been shown that in patients with COPD, the disturbed function of the vagal nerve innervating the airway can contribute to the increased secretion of BDNF and further intensify airway inflammation, thus affecting the pulmonary capacity [38]. During prolonged smoking exposure, a large amount of reactive oxidants induce processes in the lungs, leading to inflammation in the trachea and the main bronchi as well as the small airways and lung parenchyma [39]. Thus, the levels of produced and secreted BDNF may depend on the stage of the disease. Previously, a stage-dependent association of BDNF with lung function in stable COPD was reported [16]. The authors reported significantly elevated BDNF in GOLD stages (II–IV) with a strong positive correlation with forced expiratory volume in 1 s (FEV1) in all stages. Another study on the possible contribution of secreted neurotrophins on the effects of cigarettes in the airways showed that BDNF can have both short-term and long-term cigarette smoke-produced consequences on airway smooth muscles function [27]. Similarly, we found that in our studied group, non-smoking patients in GOLD stage II had significantly increased BDNF serum levels compared to the advanced III + IV stages. One possible explanation is that the developing emphysematous destruction during the progression of the disease could be a factor modulating the secretion of BDNF and the highest levels of BDNF might be achieved in patients with less severe disease [16].

## 5. Conclusions

Overall, the results of our study suggest that the expression and secretion of BDNF are changed in COPD, but its effects may differ in lung functions and COPD progression according to the smoking history of the patients. Although with several strong limitations of the current study, our results propose the notion of a protective role of BDNF in the lung function in non-smokers, while a completely opposite negative role of BDNF might be inserted on the lung tissue in patients with a history of smoking. The obtained results might shed light on the pathogenesis of airway impairment during COPD development in individuals with different cigarette smoke history. One of the most important limitations of our study is the limited number of control individuals with gender and age difference from the patients and the relatively small number of patients with COPD. That is why further studies with larger groups of controls and patients followed for some periods after inclusion in the study are necessary in order to clarify the functions of BDNF in the pathogenesis of COPD and to explore its possible role as a biomarker for the progression of this disease.

## Figures and Tables

**Table 1:** Demographic and clinical data of patients with COPD and control individuals.

Data	Patients with COPD *n* (%)	Controls *n* (%)
Number	(*n* = 57)	(*n* = 19)
Male	42 (73.7)	8 (42.1)
Female	15 (26.3)	11 (57.9)
Age at inclusion in this study		
Mean ± SEM (years)	67.14 ± 1.31	55.89 ± 2.51
Median (range) (years)	66.00 (40–88)	56.00 (35–80)
Age at diagnosis		
Mean ± SEM (years)	62.47 ± 1.44	
Median (range) (years)	64.00 (30–86)	
Duration of the disease		
Mean ± SEM (years)	4.69 ± 0.81	
Median (range) (years)	2.00 (0–30)	
Smoking status		
Non-smokers	16 (28.1)	11 (57.9)
Ex-smokers	32 (56.1)	3 (15.8)
Current smokers	9 (15.8)	5 (26.3)
Smoking habits (packs/year)		
Mean ± SD (years)	31.90 ± 2.75	14.17 ± 3.96
Median (range)	30.00 (5–88)	12.50 (5–30)
COPD stage		
GOLD II	36 (63.1)	
GOLD III	16 (28.1)	
GOLD IV	5 (8.8)	
FEV1% predicted		
Mean ± SEM	53.30 (±2.16)	
FEV1/FVC%		
Mean ± SEM	63.32 (±0.95)	

## Data Availability

Data will be made available on request from the corresponding author.
